# Electrophysiological Evaluation of Post-Activation Potentiation/Post-Activation Performance Enhancement Using Strength-Duration Properties

**DOI:** 10.3390/jfmk11020188

**Published:** 2026-05-09

**Authors:** Philip Gallardo, Antonios Papageorgiou, Vasileios Tsagkogiannis, Panagiotis V. Tsaklis

**Affiliations:** 1 ErgoMechLab, Department of Physical Education and Sport Science, University of Thessaly, 421 00 Trikala, Greece; pgallardo@uth.gr (P.G.); antpapageorgiou96@gmail.com (A.P.); tsagovasilis@gmail.com (V.T.); 2 Department Molecular Medicine and Surgery, Karolinska Institute, 171 76 Solna, Sweden; 3 TC for Safety and Health, International Ergonomics Association (IEA), P.O. Box 1226 Geneva, Switzerland

**Keywords:** post-activation potentiation, electrophysiology, electrodiagnosis, strength-duration properties, post-activation performance enhancement

## Abstract

**Background**: Strength-Duration (S-D) assessment is commonly used in clinics to examine the excitability of peripheral nerves and muscles. Yet, how changes in neuromuscular excitability relate to improved athletic and muscular performance in healthy subjects remains poorly understood. Therefore, the aim of the study was to evaluate the electrophysiological changes in neuromuscular excitability in the vastus medialis (VM) muscle using the S-D assessment, following a back squat conditioning activity (BS-CA) protocol designed to elicit a post-activation potentiation (PAP)/post-activation performance enhancement (PAPE) effect in healthy athletic males. **Methods**: Eleven male physical education students were included in this study. All subjects performed two trials: one examining their BS one-repetition maximum (1-RM), and a main experiment. During the main experiment, baseline levels of rectangular rheobase (R-RIC), triangular rheobase (R-DIC), and chronaxie were collected from the VM muscle following a standard warmup. Subsequently, the subjects performed four warmup BS sets and executed a top set of five repetitions (reps) at 80% of 1-RM. Afterwards, R-RIC, R-DIC, and chronaxie were reassessed for pre and post analysis. Based on these S-D curve (SDC) parameters, the muscle adjustability quotient (MAQ) and threshold charge (Q) were also computed and compared. **Results**: The R-RIC, R-DIC and Q were all significantly higher following the BS-CA, compared to pre-intervention (*p* < 0.001). No significant differences were observed for the chronaxie and MAQ (*p* > 0.05), although an increasing trend was noted for the chronaxie (*p* = 0.054). **Conclusions**: Based on the findings from this study, the neuromuscular excitability in the VM muscle can be acutely altered following a BS-CA protocol. However, these changes seem to be more related to muscle fatigue than PAP/PAPE. Nevertheless, S-D assessment may broaden our understanding of the fatigue process during exercise.

## 1. Introduction

It is well established that the contractile force of skeletal muscles depends on proper synaptic transmission at the neuromuscular junction (NMJ) and various membrane characteristics [1,2,3]. They regulate the initiation and propagation of action potentials, which in turn control calcium ion (Ca^+^) release from the sarcoplasmic reticulum (SR), the formation of cross-bridge cycles, and ultimately contractile force [1,4,5]. Alterations in the neuromuscular/membrane excitability, such as a higher threshold current and a reduced availability of voltage-gated sodium channels (VGSCs), have been demonstrated to impair action potential generation and transmission along the sarcolemma and T-tubules [6,7,8]. This in turn impedes the excitation–contraction coupling process, reducing SR Ca^+^ release [7,8], and consequently actin–myosin cross-bridge formation, and the production of muscle force [6,7].

Several neuromuscular disorders are linked with NMJ dysfunction and alterations in membrane function, resulting in muscle weakness [9,10,11], disability [12,13,14], and reduced quality of life [15,16]. Electrodiagnostic measures, such as evaluating strength-duration (S-D) properties, have been used as feasible, non-invasive tools to examine neuromuscular excitability and related aspects of membrane function in clinical settings [15,17,18], and as part of electrotherapy for rehabilitation [19]. Two of the most common S-D curve (SDC) parameters are the rheobase and chronaxie [17,19], often denoted by I_rh_ and τ_ch_, respectively [20,21]. The rheobase is normally defined as the minimum current required to elicit an action potential with a stimulus of infinite (or very long) duration, sometimes referred to as rectangular rheobase (R-RIC) [22,23]. The chronaxie, in contrast, refers to the minimum duration required to excite the tissue using a current twice the R-RIC [23,24]. Depending on the evaluation procedure and objective, the triangular rheobase (R-DIC) may also be examined, defined as the minimal peak current of a linearly increasing (ramp) stimulus required to elicit a detectable response [18,22].

These SDC parameters provide us with a quick overview of the excitability of peripheral nerves and muscles, including different neuromuscular disturbances [17,18,25] that can impair a patient’s movement quality and overall muscle strength [11,14]. For a broader understanding of the electrophysiology of motor nerves and skeletal muscles, the threshold current and accommodation quotient or muscle adjustability quotient (MAQ) can also be computed [20,23,25]. The threshold current, referring to the minimum current needed for excitation at a given duration [26], provides insights into the excitability of nerves/muscles [27,28,29] and can be used to monitor physiological states (i.e., fatigue and electrolyte imbalances) [28,29,30].

Different mathematical equations can be used to calculate the threshold current [20,26], but Weiss’ equation has been considered to be one of the best to explore longer and shorter stimuli currents [20], commonly referred to as the threshold charge (Q) [25,31,32]. Similarly, the MAQ helps clinicians/researchers understand a membrane’s accommodation capacity (i.e., how a membrane responds to currents rising slowly compared to abruptly) [18,23], which is useful for evaluating if a muscle is healthy or paretic. Furthermore, while these SDC parameters have been used in clinical [17,26] and rehabilitations settings [19,22], to the best of our knowledge, there is lack of data regarding how these parameters are influenced by physical activity in healthy subjects, especially exercise protocols designed to optimize athletic and muscular performance.

It is for instance well known that a warmup can enhance muscular performance via increased blood flow [33,34], enhanced motor unit discharge rates [35,36], increased speed of nervous impulses [33,34], and improved contractile force via muscle potentiation [34,37], often referred to as post-activation potentiation (PAP) [34,37,38,39]. Historically, and originally, the PAP phenomenon was confirmed by assessing the maximum twitch force, or peak twitch torque (PTT), elicited by supramaximal electrical stimulation [38], with the increased expression of myosin regulatory light chain (MRLC) phosphorylation being believed to be the primary mechanism [38,40,41]. Mechanistically, a higher expression of MRLC phosphorylation causes structural changes in the myosin heads (such as improving their mobility), allowing them to move closer to the actin binding sites [4,38].

This sequentially allows the same SR Ca^+^ release (during a contraction) to produce a greater number of active cross-bridges, enabling more force production for the same Ca^+^ concentration (i.e., increased Ca^+^ sensitivity) [4,37], ultimately enhancing the rate of force development (RFD) [4,38]. Thus, although, research and applied professionals have employed several warmup strategies with the goal of optimizing the PAP effect in athletic populations [37,39], there conflicting data has emerged regarding how changes in neuromuscular excitability modulate the PAP phenomenon [42,43]. For example, an experimental study by Hodgson et al. [42] demonstrated that the compound muscle action potential (M-wave) amplitude (reflecting changes in sarcolemmal excitability) in the soleus was acutely increased following a warmup strategy or conditioning activity (CA), consisting of a set of plantar flexion isometric maximum voluntary contractions (MVC) combined with explosive plantar flexion in resistance-trained male subjects. Notably, this M-wave enlargement coincided with the highest PTT enhancement observed 2–30 s post-CA (i.e., a PAP response).

Although this transient rise in M-wave size has been noted by several researchers [42,43,44] in different athletic populations, and even been coined M-wave potentiation [43,45], its interpretation and physiological meaning has been largely distinct [6,43,46]. For instance, some have postulated that it may, at least partially, contribute to the PAP response [44,47], being a simple motion artifact from the electrodes [43], and others have proposed that it may be related to muscle fatigue [46]. However, it is also equally common to see a largely unaffected and relatively stable M-wave, while different muscle performance outcomes are acutely enhanced [40,48,49]. Part of these differences could be related to how the PAP response was defined, as improvements in stimulated muscle contraction can occur independent of changes in voluntary contraction [38].

The PAP response has been found to be highest immediately after an isometric MVC-CA protocol, and drops exponentially over time [38,40,43], while acute improvements in voluntary muscular performance usually peak 5–10 min after the CA [38,47]. Hence, in recent years the term post-activation performance enhancement (PAPE) has more commonly been used to describe acute improvements in voluntary muscular performance following different CAs [41,50], especially when the PAP response is not directly confirmed with a twitch verification test [38,50].

However, a classical CA protocol that has been postulated to elicit both a PAP and PAPE response during different ballistic movements is heavy loaded back squats (BS) [51,52,53].

For instance, Mina and colleagues [52] observed acute improvements in peak power output (PPO) and RFD during a countermovement jump (CMJ) 30 s to 12 min after a BS-CA protocol in resistance-trained male subjects, specifically when using variable resistance.

Furthermore, although several studies have actually evaluated the M-wave in various muscles when measuring the PAP response [42,48,49], to the best of our knowledge, there is limited data regarding how it relates to PAPE.

For example, Zagatto et al. [54] observed no changes in the M-wave amplitude in the vastus lateralis muscle, while improvements were noted in a repeated sprint ability (RSA) test (i.e., a PAPE outcome) following a drop jump (DJ) CA protocol compared to a control (no exercise) group of young basketball players. However, the M-wave amplitude was examined after the RSA test in this study, and not after the DJ-CA protocol, which may have impacted the findings. While it is well established in the sports physiology literature that some accumulation of fatigue-related biomarkers is essentially inevitable during exercise [55,56,57], especially as the duration or intensity increases [55,56,58,59], the goal of a CA protocol is to maximize the benefits of PAP/PAPE while limiting the attenuating effects from muscle fatigue [58,60,61].

Based on previous literature, the PTT (i.e., PAP) and the M-wave potentiation phenomenon have both been shown to be highest immediately following an MVC-CA protocol in healthy athletic populations [42,43,44], and acute improvements in PPO and RFD during a CMJ (i.e., PAPE) have also been noted 30 s after a BS-CA protocol in resistance-trained male subjects [52], suggesting that changes in neuromuscular excitability may modulate the PAP/PAPE phenomenon, at least shortly after the CA protocol. However, a logistical constraint with accurate M-wave assessment is that the equipment required (incl. the surface electromyography system) can be expensive [62] and is less portable compared to electrodiagnostic stimulators used to examine neuromuscular excitability via S-D properties.

Having a greater understanding of how electrophysiology relates to PAP/PAPE, and whether electrodiagnostic stimulators can be used, may not only help us optimize performance and reduce unnecessary muscle fatigue in athletes, but also reduce musculoskeletal injuries, and thus a large economic burden. According to demographic data, it has been estimated that musculoskeletal injuries provide an economic burden of roughly $980 billion per year in the US [63]. Clinically, this may also be of great value for evaluating the rehabilitation process after injury. Thus, the aim of the study was to evaluate the electrophysiological changes in neuromuscular excitability in the vastus medialis (VM) muscle using the S-D assessment following a BS-CA protocol designed to elicit a PAP/PAPE effect in healthy athletic males.

## 2. Materials and Methods

### 2.1. Subjects

Eleven athletic male physical education students volunteered for this pilot study (see Table 1) with no known history of neurological or musculoskeletal impairments. All subjects had at least two years of resistance training experience, and were given oral and written explanations of the testing procedures. They also signed a written consent prior to volunteering. Furthermore, all subjects were instructed to avoid strenuous exercise, alcohol, and stimulants for at least 72 h prior to testing. This study was conducted as part of a larger research project approved by the Bioethics committee of the Department of Physical Education and Sports Science at the University of Thessaly (protocol code: 2091 and approval date: 8 February 2023). The present sub-study falls within the scope of the original ethical approval, with the study conducted in accordance with the Declaration of Helsinki.

### 2.2. Experimental Design

#### 2.2.1. Overview

A within-subjects design was used to assess differences in neuromuscular excitability in the vastus medialis (VM) muscle before and after a PAP/PAPE-inducing BS-CA protocol, using an electrodiagnostic stimulator (ELETTRONICA PAGANI™, Paderno Dugnano, Italy). Prior to the main experiment, anthropometrics were collected, in addition to evaluating the subjects’ maximum strength (one-repetition maximum (1-RM)) on the back squat (BS). This was separated by at least 72 h from the main experiment to minimize the effects of neuromuscular fatigue [64]. During the main experiments, the participants performed four low- to medium-intensity warmup BS sets and executed a top set of five repetitions (reps) at 80% of 1-RM. This has been demonstrated to be heavy enough to induce a potent PAP/PAPE effect [65,66]. Additionally, R-RIC and R-DIC was measured from the VM muscle, pre- and post-BS intervention (see Table 2).

#### 2.2.2. One Repetition Maximum Back Squat Assessment

The 1-RM BS protocol was adopted from Mina and colleagues [52]. The subject initially performed a standard warm-up using a stationary bike (Monark 874E, Varberg, Sweden) at 65 rpm with a 1 kg load for 5 min, followed by one set of 10 bodyweight (BW) squats and a BS set of 10 reps using a standard 20 kg Olympic bar, respectively. The subjects then completed a BS set of 5–6 reps at 50% of their estimated 1-RM load, before the load was increased by 10–20% for 3–5 reps, and by a further 10–20% for 2–3 reps with a 2–3 min rest interval between sets.

The final load was increased by 10%. If the set felt easy and the subjected maintained strict form, 5% was added for each consecutive 1-RM attempts, until failure or a challenging set was reached, resting 3–5 min between attempts. The heaviest successful attempt was recorded as their 1-RM squat load. To control the technique, subjects were instructed to place the bar above the posterior deltoids at the lower neck region (around C7 level) and attempt to squat to a position where the knee was flexed to ~90° before returning to a standing position. This was visually assessed by a coach with an Olympic weightlifting certification to ensure safe and correct lifting technique.

#### 2.2.3. Main Experiment—Back Squat Protocol

During the main experiments the subjects performed a task-specific warm-up consisting of 5 min of cycling, and pre-intervention measures of SDC parameters (see Table 2). This was followed by one set of 10 BW squats and a BS set of 10 reps using a standard 20 kg Olympic bar, respectively. Subsequently, three additional low to moderate BS sets were performed at 50%, 60–65%, and 70–75% of the previously determined 1-RM load for six, five, and five reps respectively (see Table 3). The last and top set was performed at 80% of 1-RM for five reps.

#### 2.2.4. Collection of Rheobase Parameters and the Chronaxie

An electrodiagnostic stimulator was used to examine neuromuscular excitability in the VM muscle via S-D properties. The skin was shaved, abraded, and cleaned with alcohol, prior to placing bipolar adhesive surface electrodes (Noraxon Dual Electrodes, Ag/AgCL snap, Noraxon USA, Inc, Scottsdale, AZ, USA) on the belly of the VM muscle. Specifically, the anode (reference electrode) was placed over the proximal region of the anterior thigh, proximal to the motor point of the VM. The cathode (active electrode) was placed directly over the distally located motor point of the VM, corresponding to the region of the VM, as previously described by Botter and colleagues [67].

For the rheobase measurement, the R-RIC was assessed before and immediately after the squat-intervention using a 1000 ms duration square-wave current pulse, while R-DIC was assessed using a 1000 ms duration triangular-wave current pulse (linearly increasing), respectively. In both rheobase conditions, the stimuli were separated by a 2 s inter-stimulus (rest). Furthermore, the current increased from 0 to 35 mA in 1 mA increments until a slight but apparent muscle contraction was visible. For the chronaxie, the pulse duration was decreased from 0.1 ms to 0.5 ms in 0.05 ms increments, until a consistent visible muscle contraction was observed.

### 2.3. Formulas for Threshold Charge and Muscle Adjustability Quotient

The threshold current was calculated using Weiss formula, corresponding to the threshold charge (Q) as described by Weiss’s law. The Q represents the minimal charge delivered at the given stimulus duration needed to elicit an action potential or observable muscle contraction. Weiss formula is often calculated using the following equation [31].

**Figure 1:**
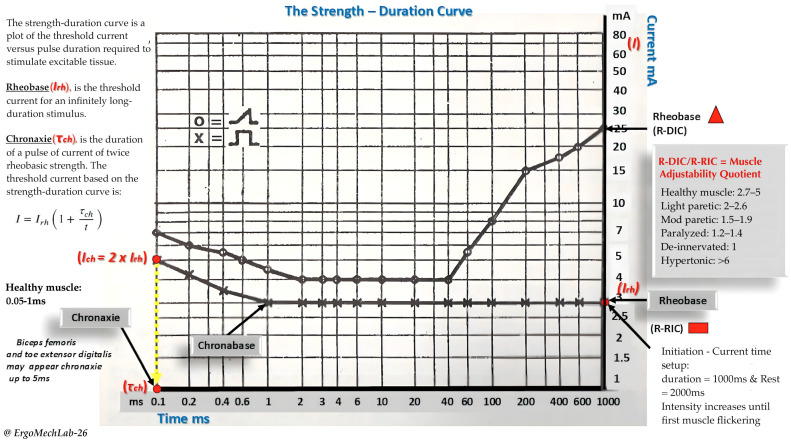
This figure provides an overview of the strength–duration curve and includes some reference values for the chronaxie and the muscle adjustability quotient, respectively. The O and X symbols in this figure describes the curve of different nerve/muscle conditions. The O denotes the curve of a partially denervated muscle, while X represents the shape of a healthy and normal muscular response.

Q = I_rh_ (t + τ_ch_)

where I_rh_ is the R-RIC in mA, t is the stimulus duration, and τ_ch_ is the chronaxie, in ms, respectively. The Q is accordingly expressed in mA ms, corresponding to microcoulomb (μC) which is a standard unit of electric charge (Kloth 2014) and commonly used in electrophysiology studies [26,68,69]. In contrast, the muscle adjustability quotient (MAQ) was determined from the R-RIC and R-DIC measurements to evaluate the accommodation properties of the neuromuscular system. This ratio reflects the ability of the membrane to respond to currents rising slowly (triangular) compared to abruptly (rectangular) [18,23]. MAQ was calculated using the following equation (See Figure 1).

$$\mathrm{MAQ}=\frac{R-DIC}{R-RIC}$$

### 2.4. Statistical Analysis

The data are presented as means ± standard deviations (SD) unless otherwise stated. The differences between pre-intervention and post-intervention were normally distributed and were checked using the Shapiro–Wilk test. Paired *t*-tests were carried out to compare the means of each SDC parameter, respectively. Effect sizes (Cohen’s *d*) were calculated to characterize the magnitude of the observed differences and were interpreted following conventional guidelines: 0.2 = small, 0.5 = medium, 0.8 = large. All statistical analyses were executed using SPSS v.31.0 statistical program for MacOS (SPSS Software, IBM Inc., Chicago, IL, USA). The level of significance was set at *p* < 0.05.

## 3. Results

### 3.1. Rheobase Parameters and the Chronaxie

**Figure 2:**
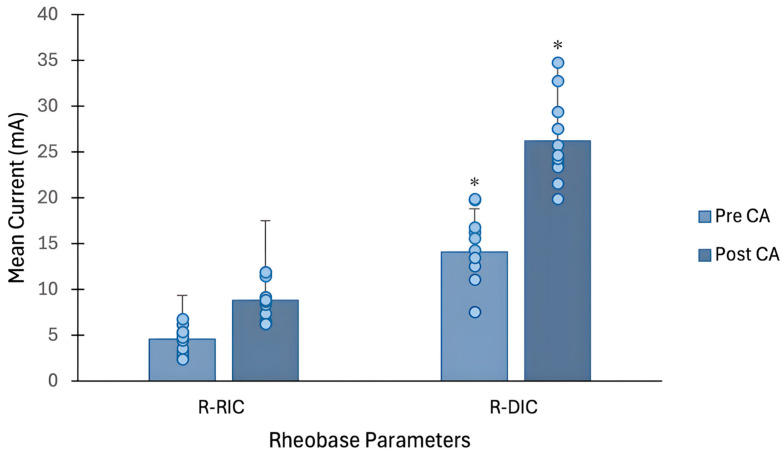
A bar graph (with subject data points) illustrating differences in rheobase parameters following the back squat protocol. * = *p* < 0.001 compared to pre-intervention.

**Figure 3:**
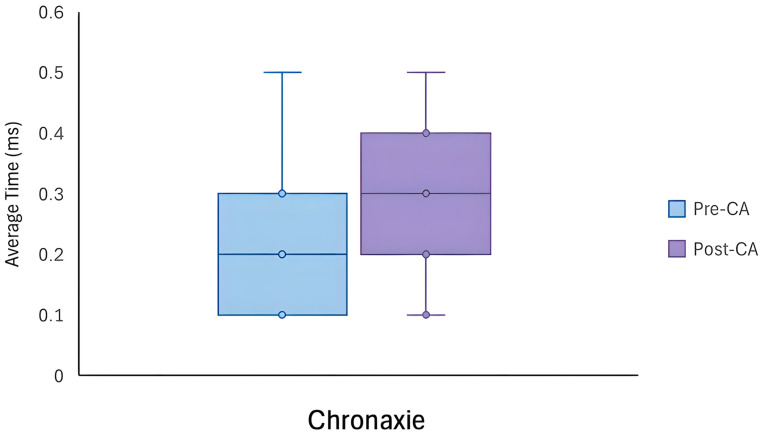
A box plot demonstrating a trend towards a higher chronaxie value following the back squat protocol.

There was a significant difference in all rheobase parameters after the BS-CA protocol. Specifically, the R-RIC was significantly higher following the BS intervention (M = 8.83, SD = 2.15) compared to pre-intervention (M = 4.56, SD = 1.45), t(10) = −9.150, *p* < 0.001, *d* = 2.8. Similarly, a higher R-DIC was noted following the BS-CA protocol (M = 26.18, SD = 4.6) compared to pre-intervention (M = 14.01, SD = 4.2), t(10) = −7.037, *p* < 0.001, *d* = 2.1 (see Figure 2). Although no significant differences were observed between the chronaxie values pre- and post-intervention, a trend was observed (see Figure 3). In particular, following the BS intervention, the mean chronaxie was trending towards a higher value (M = 0.27, SD = 0.13) compared to pre-intervention (M = 0.20, SD = 0.13), t(10) = −2.19, *p* = 0.054, *d* = 0.66 (see Table 4).

### 3.2. Threshold Charge and Muscle Adjustability Quotient

**Figure 4:**
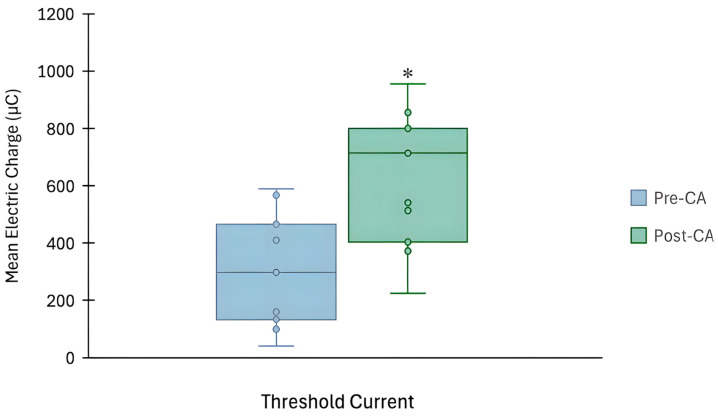
A box plot (with subject data points) revealing differences in threshold charge following the back squat protocol. * = *p* < 0.001 compared to pre-intervention.

No significant differences were observed for the MAQ post-intervention (M = 3.05, SD = 0.54) compared to pre-intervention (M = 3.12, SD = 0.34), t(10) = 0.53, *p* = 0.61, *d* = 0.16. Interestingly, however, there was a significant change in the Q pre- and post-BS-CA protocol (see Figure 4). Specifically, the mean Q was notably higher in the post-squat intervention (M = 627.36, SD = 231.13) compared to pre-intervention (M = 293.14, SD = 192.78), t(10) = −4.48, *p* < 0.001, *d* = 1.4 (see Table 5).

## 4. Discussion

The purpose of this study was to evaluate the electrophysiological changes in neuromuscular excitability in the VM muscle, using the S-D assessment, following a BS-CA protocol designed to elicit a PAP/PAPE effect in healthy athletic males. The findings from our study suggest that the neuromuscular excitability of the VM can be acutely altered, following a standard PAP/PAPE-inducing CA protocol. Paradoxically, however, our study revealed that rheobase parameters (i.e., R-RIC and R-DIC) and the Q in the VM muscle increased after the BS intervention (see Figure 2 and Figure 4), indicating reduced neuromuscular excitability [70] and presumably muscle fatigue [28]. While no differences were observed for the chronaxie, there was a trend towards a higher chronaxie in the VM following the BS intervention (see Figure 3), pointing towards slower membrane dynamics and responsiveness [24]. Interestingly, however, the MAQ remained stable throughout the intervention, implying that the membrane accommodation properties were still preserved [18].

This is in line with earlier studies suggesting that neuromuscular excitability and related aspects of membrane function are not directly involved with the PAP/PAPE phenomenon [48,49,71] but contradicts the notion that neuromuscular excitability are not altered after a CA protocol designed to induce PAP/PAPE [71,72]. Previous research has consistently shown that sarcolemma excitability (via recording M-waves) in the VM [49], but also in the vastus lateralis [48] and soleus [40], tend to remain stable after a CA protocol while there is an observable improvement in twitch force [48,49] or voluntary performance outcomes [40,48] (i.e., PAP and PAPE, respectively) in different athletic populations, implying that acute improvements in muscular performance can occur independent of evident changes in neuromuscular excitability.

Intriguingly, however, a transient ‘M-wave enlargement’ has also been noted by several researchers following different CA protocols [42,43], which has commonly been referred to as M-wave potentiation [43,45]. Although there has been mixed interpretation about this M-wave phenomenon [6,43,44], it is clear that it can also occur following a fatigue-inducing exercise protocol [6,73]. An experimental study by Bigland-Ritchie et al. [73] demonstrated that a continuous MVC protocol of the adductor pollicis muscle for 1 min impaired force by 40–60% while the M-wave size (elicited via a single supramaximal stimulus) increased.

Furthermore, more recently it has been suggested that an increase in M-wave size may in fact reflect excitability disruption via prolonged transmembrane potential [74,75]. This broadening of the intracellular potential has been demonstrated to be related to increased extracellular potassium concentrations ([K^+^]_0_) [74,76], which are elevated during intense exercise and are believed to be a major cause of muscle fatigue [6,77,78]. Thus reduced neuromuscular excitability, which can be presumed through a higher R-RIC, R-DIC, and Q, can clearly be a sign of muscle fatigue. Furthermore, based on the work of Sale [58], it has been well recognized for decades that the PAP/PAPE effect and muscle fatigue normally coexist, and that the dissipation of fatigue needs to be greater than the decay rate of PAP/PAPE for the involved muscles to be in a net potentiated state [58,60,61]. Hence, although it is unclear if the CA protocol in our study resulted solely in more fatigue than muscle potentiation in the VM muscle, earlier research suggested that M-wave potentiation can coincide with both PAP/PAPE [42,44] and muscle fatigue [6,74], implying that it is still possible to observe markers of fatigue (e.g., reduced neuromuscular excitability) following a CA protocol, independent of any evident PAP/PAPE effect.

Therefore, while evaluating S-D properties may provide minimal mechanistic insights into the PAP/PAPE phenomenon, they may broaden our understanding of the fatigue process. For instance, a recent pharmaceutical study by Rocchi et al. [79] demonstrated that taking sodium channel blockers, such as lacosamide, consistently raised rheobase levels relative to a placebo or carbamazepine. The authors in this study proposed that this was due to the blocking action on VGSCs, which earlier works have noted are critical for regulating axonal excitability [80,81]. Additionally, an ex vivo experiment demonstrated that tetrodotoxin (a potent neurotoxin) directly inhibited several sodium channels, including Na_v_1.7, and this was linked with a higher rheobase, reduced neuronal firing rate, and reduced nociceptor excitability [82]. Furthermore, during intense fatiguing muscular activity, it is also well established that [K^+^]_0_ not only increases, but there is also a reduction in intracellular K^+^ concentration ([K^+^]_i_), which together (via the K^+^ gradient) depolarize the resting membrane potential [78]. Based on in vitro experiments, depolarization can sequentially reduce the availability of VGSCs by promoting channel inactivation [83,84]. This reduction in available VGSCs leads to a decrease in sodium current [83], which has been shown to be reflected by an increased rheobase [85], Q [86] and a reduction in overall neuromuscular/membrane excitability [6,83,85,86].Thus, although not directly evaluated, we can postulate that the BS-CA protocol may have been adequately fatigue-inducing to acutely raise [K^+^]_0_ and reduce [K^+^]_i_, and this consequently increased the R-RIC, R-DIC, and Q in the VM in our study (see Figure 2 and Figure 4).

Additionally, based on previous studies, a higher rheobase and Q is also evidence of a higher electric impedance [87] and signifies that a greater amount of electrical input is required to reach the depolarization threshold [31,86], both resulting in reduced neuromuscular/membrane excitability [86,87]. While no changes were observed for the chronaxie, the trend pointed towards a higher value following the BS-CA protocol, inferring a slower membrane response [24]. Although it is less clear how the inactivation of VGSCs relates to the chronaxie, experimental data grounded in in vitro studies suggests that the chronaxie can also be altered, at least when using cultured hippocampal neurons from rats [88].

These discrepancies in our findings can in part be attributed to how the chronaxie were collected or computed (e.g., extracting it from the SDC vs. equating it to the membrane time constant) and differences in cellular geometry [88,89]. Interestingly, we also found that the MAQ were unaffected by the BS-CA protocol, implying that the membrane accommodation properties were still preserved [18]. Based on previous literature [18,23], the subjects in our study were within the lower of end of the healthy range (for reference, see Figure 1) on average (see Table 5), pointing towards more excitable tissues than the general population.

As the subjects in our study were healthy, athletic, and resistance-trained, and most in vivo human research (using electrodiagnostic tests) has been conducted on patients with neuromuscular disorders [17,19,23], this would be expected. Interestingly, although the subjects in our study were considered overweight according to population-based BMI standards [90,91], a BMI between 25 and 30 kg/m^2^ is generally not indicative of excess adipose tissue in athletic populations [91,92], and is more frequently indicative of a higher lean body mass [92,93]. Since most subjects in our study squatted 1.7 times their BW on average, and some even reached twice their BW (see Table 1), which are displays of strength more commonly observed in elite strength-power athletes [94,95,96,97], we can reasonably presume that the higher BMI of our subjects was attributed to a higher proportion of lean body mass, even if body composition was not directly examined.

Still, however, a key limitation of this study is that twitch force (i.e., PAP) and voluntary muscular performance outcomes (i.e., PAPE) were not directly evaluated. Importantly, earlier data suggest that PAP is usually highest immediately after the CA protocol [42,43,44], and the M-wave potentiation phenomenon (i.e., acute changes in the membrane excitability of skeletal muscles) has been shown to be very short-lived (≤1 min) [44], hence why the primary focus of this study was to collect the S-D data immediately following the BS-CA protocol.

Moreover, while our study suggests that neuromuscular excitability can be acutely altered in the VM muscle, following a standard CA protocol designed to induce PAP/PAPE, another limitation is that the underlying physiology cannot be fully characterized by S-D properties alone. For instance, evidence from in vitro experiments suggests a reduced neuromuscular/membrane excitability is mediated by increases in membrane capacitance and decreases in membrane resistance [98,99]. However, the relative contribution of these mechanisms cannot be clearly delineated without including more advanced procedures. Furthermore, another notable limitation with S-D assessment is that it almost exclusively reflects neuromuscular excitability at the peripheral level (i.e., muscles and peripheral nerves) [32,100], as the central nervous system (CNS) is effectively bypassed during electrical stimulation [101].

Hence, to examine the involvement of the CNS, S-D properties need to be complemented with techniques such as electroencephalogram (EEG) [102], or even more noteworthy transcranial magnetic stimulation (TMS) [79], a procedure that allows measures of excitability within the motor cortex and corticospinal pathways [79,103]. Similarly, although SDC parameters offer insight into excitability at the peripheral neuromuscular level, alterations in Ca^+^ sensitivity (an intramuscular mechanism contributing to the PAP phenomenon) cannot be inferred by SDC parameters alone.

### 4.1. Practical Applications

The results from this paper suggest that measuring S-D properties may provide us with new insights into the fatigue process during exercise, in particular the development of fatigue following warm up strategies designed to optimize muscular performance. Thus, in a sports context, this may help coaches get a broader understanding of the individual fatigue profile of an athlete. Additionally, tracking different SDC parameters (especially the R-RIC and R-DIC) in an injury-free and non-fatigue state may help coaches and athletes to evaluate the recovery process after injury. Due to its accessibility and non-invasive nature, it may also have some utility for examining training adaptations following different exercise protocols. Thus, to understand the potential use of electrodiagnostic stimulators in sports settings, more studies are clearly warranted.

### 4.2. Limitations and Future Recommendations

The present study has several methodological limitations. This includes the small sample size and lack of control condition, and the S-D properties which were only assessed at one time point and did not use a longitudinal design or include a mixed-method.

This limits the generalizability of the results. Further studies should therefore include a larger sample size, include a control condition, include more time intervals, and use a more robust study design to improve our understanding of how neuromuscular excitability and related membrane functions modulate the PAP/PAPE phenomenon. Additionally, to determine the contribution of the CNS, further studies should consider adding procedures such as EEG and/or TMS and use a range of different CA protocols. Having a greater understanding of how changes in neuromuscular excitability relate to PAP/PAPE may help us reduce muscle fatigue, and thus injury risk. According to demographic data, it has been estimated that musculoskeletal injuries provide an economic burden of roughly $980 billion per year in the US. This paper therefore highlights the potential value of understanding how electrophysiological changes in neuromuscular excitability relate to the PAP/PAPE phenomenon.

## 5. Conclusions

This study aimed to evaluate the electrophysiological changes in neuromuscular excitability in the VM muscle, using the S-D assessment, following a BS-CA protocol designed to elicit a PAP/PAPE effect in healthy athletic males. The findings demonstrated that a standard BS-CA protocol can acutely increase rheobase parameters (R-RIC and R-DIC, respectively), the Q, and possibly the chronaxie (based on the observed trend).

These preliminary findings demonstrate reduced neuromuscular excitability and presumably muscle fatigue, implying that evaluating S-D properties may provide us with new insights into the fatigue process during exercise.

However, due to the sample size, these findings cannot be generalized. Hence, this study should be replicated with a larger sample and include different subgroups (e.g., athletic female subjects). Additionally, since the S-D assessment primarily reflects neuromuscular excitability within muscles and peripheral nerves, future studies should also employ different procedures that control for excitability within the motor cortex and corticospinal pathways. Having a greater understanding of this may not only help us optimize performance and reduce unnecessary muscle fatigue, but also reduce musculoskeletal injuries, and thus a large economic burden. Clinically, this may also be of great value for evaluating the rehabilitation process after injury. This paper therefore highlights the potential value of understanding how electrophysiological changes in neuromuscular excitability relate to the PAP/PAPE effect.

## Figures and Tables

**Table 1:** Characteristics of the subjects (*n* = 11).

Characteristics	(Mean ± SD)
Age (years)	19.6 ± 1.5
Height (cm)	175.6 ± 3.5
Weight (kg)	78.3 ± 13.8
1-RM BS (kg)	135.45 ± 40.4
Rel. 1-RM BS (kg·kg^−1^)	1.71 ± 0.3
BMI (kg/m^2^)	25.34 ± 3.73

SD = Standard Deviation; 1-RM = One Repetition Maximum; BS = Back Squats; Rel. = Relative; BMI = Body Mass Index.

**Table 2:** Timeline for the main experiment.

Task	Time (min)
Preparation (skin, electrodes and machine)	0–5.0
5 min cycling	5.5–10.5
Pre-Intervention (R-RIC, R-DIC and τ_ch_)	11.0–13.0
10 BW Squats	13.5–14.5
BS-CA Protocol	15.0–29.0
Post-Intervention (R-RIC, R-DIC and τ_ch_)	29.5–31.5

R-RIC = Rectangular rheobase; R-DIC = Triangular rheobase; τ_ch_ = Chronaxie; BW = BW = Body weight; BS-CA = Back squat conditioning activity.

**Table 3:** A BS-CA protocol for eliciting a PAP/PAPE effect.

Intensity (% of 1-RM)	Reps	Rest (min)/Set
<50 (20 kg)	10	2
50	6	2
60–65	5	3
70–75	5	3
80 (Top Set)	5	

BS-CA = Back squat conditioning activity; PAP/PAPE = Post-Activation Potentiation/Post-Activation Performance Enhancement; 1-RM = One repetition maximum; Reps = Repetitions.

**Table 4:** Rheobase parameters and chronaxie at baseline compared to post-intervention.

Parameter	Pre	Post	Cohen’s *d*	*p*-Value
R-RIC (mA)	4.56 ± 1.45	8.83 ± 2.15	2.8	<0.001
R-DIC (mA)	14.01 ± 4.2	26.18 ± 4.6	2.1	<0.001
τ_ch_ (ms)	0.20 ± 0.13	0.27 ± 0.13	0.66	0.054

Acronyms: R-RIC = Rectangular rheobase; R-DIC = Triangular rheobase; τ_ch_ = Chronaxie; Pre = Pre-intervention; Post = Post-intervention.

**Table 5:** Threshold charge and muscle adjustability quotient at baseline compared to post-intervention.

Parameter	Pre	Post	Cohen’s *d*	*p*-Value
Q (μC)	293.14 ± 192.78	627.36 ± 231.13	1.4	<0.001
MAQ	3.12 ± 0.34	3.05 ± 0.54	0.16	0.61

Acronyms: Q = Threshold Charge; MAQ = Muscle Adjustability Quotient; Pre = Pre-Intervention; Post = Post-Intervention.

## Data Availability

The data presented in this study are available on request from the corresponding author due to ethical considerations.

## Abbreviations

The following abbreviations are used in this manuscript:

[K^+^]_0_	Extracellular Potassium Concentrations
[K^+^]_i_	Intracellular Potassium Concentrations
1-RM	One-Repetition Maximum
BS	Back Squat
BW	Bodyweight
CA	Conditioning Activity
Ca^+^	Calcium Ion
CNS	Central Nervous System
CMJ	Countermovement Jump
DJ	Drop Jump
EEG	Electroencephalogram
M-wave	Compound Muscle Action Potential
MAQ	Muscle Adjustability Quotient
MRLC	Myosin Regulatory Light Chain
MVC	Maximum Voluntary Contraction
NMJ	Neuromuscular Junction
PAP	Post-Activation Potentiation
PAPE	Post-Activation Performance Enhancement
PPO	Peak Power Output
PTT	Peak Twitch Torque
Q	Threshold Charge
R-DIC	Triangular Rheobase
R-RIC	Rectangular Rheobase
Reps	Repetitions
RFD	Rate of Force Development
RSA	Repeated Sprint Ability
S-D	Strength-Duration
SD	Standard Deviations
SDC	Strength-Duration Curve
SR	Sarcoplasmic Reticulum
TMS	Transcranial Magnetic Stimulation
VGSCs	Voltage-Gated Sodium Channels
VM	Vastus Medialis
μC	Microcoulomb

## References

[1] Plant D.R., Lynch G.S. (2002). Excitation-contraction coupling and sarcoplasmic reticulum function in mechanically skinned fibres from fast skeletal muscles of aged mice. J. Physiol..

[2] Hernández-Ochoa E.O., Schneider M.F. (2018). Voltage sensing mechanism in skeletal muscle excitation-contraction coupling: Coming of age or midlife crisis?. Skelet. Muscle.

[3] Wang B., Dudko O.K. (2021). A theory of synaptic transmission. eLife.

[4] Stull J.T., Kamm K.E., Vandenboom R. (2011). Myosin light chain kinase and the role of myosin light chain phosphorylation in skeletal muscle. Arch. Biochem. Biophys..

[5] Pitake S., Ochs R.S. (2015). Membrane depolarization increases ryanodine sensitivity to Ca^2+^ release to the cytosol in L6 skeletal muscle cells: Implications for excitation–contraction coupling. Exp. Biol. Med..

[6] Allen D.G., Lamb G.D., Westerblad H. (2008). Skeletal Muscle Fatigue: Cellular Mechanisms. Physiol. Rev..

[7] Cairns S.P., Taberner A.J., Loiselle D.S. (2009). Changes of surface and t-tubular membrane excitability during fatigue with repeated tetani in isolated mouse fast- and slow-twitch muscle. J. Appl. Physiol..

[8] Fauler M., Jurkat-Rott K., Lehmann-Horn F. (2012). Membrane excitability and excitation–contraction uncoupling in muscle fatigue. Neuromuscul. Disord..

[9] Kaya P., Alemdaroğlu İ., Yılmaz Ö. (2015). Effect of muscle weakness distribution on balance in neuromuscular disease. Pediatr. Int..

[10] Iyer S.R., Shah S.B., Lovering R.M. (2021). The Neuromuscular Junction: Roles in Aging and Neuromuscular Disease. Int. J. Mol. Sci..

[11] Padilla C.J., Harrigan M.E., Harris H., Schwab J.M., Rutkove S.B., Rich M.M., Clark B.C., Arnold W.D. (2021). Profiling age-related muscle weakness and wasting: Neuromuscular junction transmission as a driver of age-related physical decline. GeroScience.

[12] Shigemoto K., Kubo S., Mori S. (2010). Muscle weakness and neuromuscular junctions in aging and disease. Geriatr. Gerontol. Int..

[13] Jerath N.U., Simoens K., Mann D. (2019). Survey of the functional priorities in patients with disability due to neuromuscular disorders. Disabil. Rehabil. Assist. Technol..

[14] Miao Y., Xie L., Song J., Cai X., Yang J., Ma X., Chen S., Xie P. (2024). Unraveling the causes of sarcopenia: Roles of neuromuscular junction impairment and mitochondrial dysfunction. Physiol. Rep..

[15] Kwai N.C., Arnold R., Wickremaarachchi C., Lin C.S.-Y., Poynten A.M., Kiernan M.C., Krishnan A.V. (2013). Effects of Axonal Ion Channel Dysfunction on Quality of Life in Type 2 Diabetes. Diabetes Care.

[16] Wong K.S.W., Sen A., Michell-Sodhi J. (2026). Assessing the Relationship of Quality of Life with Functional Status in a Large Cohort of Adult Patients with Neuromuscular Disorders. Neurol. Clin. Pract..

[17] Silva P.E., Maldaner V., Vieira L., de Carvalho K.L., Gomes H., Melo P., Babault N., Cipriano G., Durigan J.L.Q. (2018). Neuromuscular electrophysiological disorders and muscle atrophy in mechanically-ventilated traumatic brain injury patients: New insights from a prospective observational study. J. Crit. Care.

[18] Wróbel B., Dolibog P., Penkala A., Kierszniok K., Król P. (2022). The Determination of Normative Values for the Median Nerve Using Classic Electrodiagnostic Methods. Rehabil. Med..

[19] Lee W.-D., Kim J.-H., Lee J.-U., Kim M.-Y., Lee L.-K., Yang S.-M., Jeon H.-J., Lee T.-H., Kim J. (2013). Differences in Rheobase and Chronaxie between the Paretic and Non-Paretic Sides of Hemiplegic Stroke Patients: A Pilot Study. J. Phys. Ther. Sci..

[20] Bostock H. (1983). The strength-duration relationship for excitation of myelinated nerve: Computed dependence on membrane parameters. J. Physiol..

[21] Smit J.E., Hanekom T., Hanekom J.J. (2008). Predicting action potential characteristics of human auditory nerve fibres through modifi-cation of the Hodgkin–Huxley equations. S. Afr. J. Sci..

[22] Arnold D., Thielker J., Klingner C.M., Puls W.C., Misikire W., Guntinas-Lichius O., Volk G.F. (2021). Selective Surface Electrostimulation of the Denervated Zygomaticus Muscle. Diagnostics.

[23] De-La-Cruz-Torres B., Abuín-Porras V., Navarro-Flores E., Calvo-Lobo C., Romero-Morales C. (2021). Ultrasound-Guided Percutaneous Neuromodulation in Patients with Chronic Lateral Epicondylalgia: A Pilot Randomized Clinical Trial. Int. J. Environ. Res. Public Health.

[24] Geddes L. (2004). Accuracy Limitations of Chronaxie Values. IEEE Trans. Biomed. Eng..

[25] Salian S.C., Tulsankar G.N. (2017). Strength-duration curve: A measure for assessing pain in trapezius spasm. Indian J. Pain.

[26] Abalkhail T.M., MacDonald D.B., AlThubaiti I., AlOtaibi F.A., Stigsby B., Mokeem A.A., AlHamoud I.A., Hassounah M.I., Baz S.M., AlSemari A. (2017). Intraoperative direct cortical stimulation motor evoked potentials: Stimulus parameter recommendations based on rheobase and chronaxie. Clin. Neurophysiol..

[27] Krarup C., Moldovan M. (2009). Nerve conduction and excitability studies in peripheral nerve disorders. Curr. Opin. Neurol..

[28] Fortune E., Lowery M.M. (2012). Effect of membrane properties on skeletal muscle fiber excitability: A sensitivity analysis. Med. Biol. Eng. Comput..

[29] Boërio D., Bostock H., Spescha R., Z’GRaggen W.J. (2014). Potassium and the Excitability Properties of Normal Human Motor Axons In Vivo. PLoS ONE.

[30] Kiernan M.C., Walters R.J.L., Andersen K.V., Taube D., Murray N.M.F., Bostock H. (2002). Nerve excitability changes in chronic renal failure indicate membrane depolarization due to hyperkalaemia. Brain.

[31] Weiss G. (1901). Sur la possibilite de rendre comparables entre eux les appareils servant a l’excitation electrique. Arch. Ital. Biol..

[32] Mogyoros I., Kiernan M.C., Burke D. (1996). Strength-duration properties of human peripheral nerve. Brain.

[33] Shellock F.G., Prentice W.E. (1985). Warming-Up and Stretching for Improved Physical Performance and Prevention of Sports-Related Injuries. Sports Med..

[34] Bishop D. (2003). Warm Up I: Potential Mechanisms and the Effects of Passive Warm Up on Exercise Performance. Sports Med..

[35] Duchateau J., Baudry S. (2014). Maximal discharge rate of motor units determines the maximal rate of force development during ballistic contractions in human. Front. Hum. Neurosci..

[36] Lee J., Park D., Lee J.-Y., Park J. (2024). Effect of Warm-Up Exercise on Functional Regulation of Motor Unit Activation during Isometric Torque Production. J. Hum. Kinet..

[37] McGowan C.J., Pyne D.B., Thompson K.G., Rattray B. (2015). Warm-Up Strategies for Sport and Exercise: Mechanisms and Applications. Sports Med..

[38] Blazevich A.J., Babault N. (2019). Post-activation Potentiation Versus Post-activation Performance Enhancement in Humans: Historical Perspective, Underlying Mechanisms, and Current Issues. Front. Physiol..

[39] Garbisu-Hualde A., Santos-Concejero J. (2021). Post-Activation Potentiation in Strength Training: A Systematic Review of the Scientific Literature. J. Hum. Kinet..

[40] Miyamoto N., Kanehisa H., Fukunaga T., Kawakami Y. (2011). Effect of Postactivation Potentiation on the Maximal Voluntary Isokinetic Concentric Torque in Humans. J. Strength Cond. Res..

[41] Cuenca-Fernández F., Smith I.C., Jordan M.J., MacIntosh B.R., López-Contreras G., Arellano R., Herzog W. (2017). Nonlocalized postactivation performance enhancement (PAPE) effects in trained athletes: A pilot study. Appl. Physiol. Nutr. Metab..

[42] Hodgson M.J., Docherty D., Zehr E.P. (2008). Postactivation Potentiation of Force Is Independent of H-Reflex Excitability. Int. J. Sports Physiol. Perform..

[43] Folland J.P., Wakamatsu T., Fimland M.S. (2008). The influence of maximal isometric activity on twitch and H-reflex potentiation, and quadriceps femoris performance. Eur. J. Appl. Physiol..

[44] Hamada T., Sale D.G., MacDougall J.D., Tarnopolsky M.A. (2000). Postactivation potentiation, fiber type, and twitch contraction time in human knee extensor muscles. J. Appl. Physiol..

[45] Hicks A., Fenton J., Garner S., McComas A.J. (1989). M wave potentiation during and after muscle activity. J. Appl. Physiol..

[46] Rodriguez-Falces J., Place N. (2021). Sarcolemmal Excitability, M-Wave Changes, and Conduction Velocity During a Sustained Low-Force Contraction. Front. Physiol..

[47] Gallardo P., Giakas G., Sakkas G.K., Tsaklis P.V. (2024). Are Surface Electromyography Parameters Indicative of Post-Activation Potentiation/Post-Activation Performance Enhancement, in Terms of Twitch Potentiation and Voluntary Performance? A Systematic Review. J. Funct. Morphol. Kinesiol..

[48] Seitz L.B., Trajano G.S., Maso F.D., Haff G.G., Blazevich A.J. (2015). Postactivation potentiation during voluntary contractions after continued knee extensor task-specific practice. Appl. Physiol. Nutr. Metab..

[49] Johnson M., Baudin P., Ley A.L., Collins D.F. (2019). A Warm-Up Routine That Incorporates a Plyometric Protocol Potentiates the Force-Generating Capacity of the Quadriceps Muscles. J. Strength Cond. Res..

[50] Boullosa D. (2021). Post-activation performance enhancement strategies in sport: A brief review for practitioners. Hum. Mov..

[51] Esformes J.I., Bampouras T.M. (2013). Effect of Back Squat Depth on Lower-Body Postactivation Potentiation. J. Strength Cond. Res..

[52] Mina M.A., Blazevich A.J., Tsatalas T., Giakas G., Seitz L.B., Kay A.D. (2018). Variable, but not free-weight, resistance back squat exercise potentiates jump performance following a comprehensive task-specific warm-up. Scand. J. Med. Sci. Sports.

[53] de Freitas M.C., Rossi F.E., Colognesi L.A., de Oliveira J.V.N., Zanchi N.E., Lira F.S., Cholewa J.M., Gobbo L.A. (2021). Postactivation Potentiation Improves Acute Resistance Exercise Performance and Muscular Force in Trained Men. J. Strength Cond. Res..

[54] Zagatto A.M., Claus G.M., Dutra Y.M. (2022). Drop jumps versus sled towing and their effects on repeated sprint ability in young basketball players. BMC Sports Sci. Med. Rehabil..

[55] Enoka R.M., Duchateau J. (2008). Muscle fatigue: What, why and how it influences muscle function. J. Physiol..

[56] Finsterer J. (2012). Biomarkers of peripheral muscle fatigue during exercise. BMC Musculoskelet. Disord..

[57] Wan J., Qin Z., Wang P. (2017). Muscle fatigue: General understanding and treatment. Exp. Mol. Med..

[58] Sale D.G. (2002). Postactivation potentiation: Role in human performance. Exerc. Sport. Sci. Rev..

[59] Stastny P., Kolinger D., Pisz A. (2024). Effects of Eccentric Speed during Front Squat Conditioning Activity on Post-activation Performance Enhancement of Hip and Thigh Muscles. J. Hum. Kinet..

[60] Robbins D.W. (2005). Postactivation Potentiation and Its Practical Applicability: A Brief Review. J. Strength Cond. Res..

[61] Tillin N.A., Bishop D. (2009). Factors Modulating Post-Activation Potentiation and its Effect on Performance of Subsequent Explosive Activities. Sports Med..

[62] Del Toro S.F., Wei Y., Olmeda E., Ren L., Guowu W., Díaz V. (2019). Validation of a Low-Cost Electromyography (EMG) System via a Commercial and Accurate EMG Device: Pilot Study. Sensors.

[63] Nguyen A.T., Aris I.M., Snyder B.D., Harris M.B., Kang J.D., Murray M., Rodriguez E.K., Nazarian A. (2024). Musculoskeletal health: An ecological study assessing disease burden and research funding. Lancet Reg. Health—Am..

[64] Thomas K., Brownstein C.G., Dent J., Parker P., Goodall S., Howatson G. (2018). Neuromuscular Fatigue and Recovery after Heavy Resistance, Jump, and Sprint Training. Med. Sci. Sports Exerc..

[65] Tsoukos A., Brown L.E., Veligekas P., Terzis G., Bogdanis G.C. (2019). Postactivation Potentiation of Bench Press Throw Performance Using Velocity-Based Conditioning Protocols with Low and Moderate Loads. J. Hum. Kinet..

[66] Eken Ö., Mainer-Pardos E., Yagin F.H., Eken I., Prieto-González P., Nobari H. (2022). Motoric performance variation from morning to evening: 80% intensity post-activation potentiation protocol impacts performance and its diurnal amplitude in basketball players. Front. Psychol..

[67] Botter A., Oprandi G., Lanfranco F., Allasia S., Maffiuletti N.A., Minetto M.A. (2011). Atlas of the muscle motor points for the lower limb: Implications for electrical stimulation procedures and electrode positioning. Eur. J. Appl. Physiol..

[68] Babb T.L., Soper H.V., Lieb J.P., Brown W.J., Ottino C.A., Crandall P.H. (1977). Electrophysiological studies of long-term electrical stimulation of the cerebellum in monkeys. J. Neurosurg..

[69] Kloth L.C. (2014). Electrical Stimulation Technologies for Wound Healing. Adv. Wound Care.

[70] Rodríguez-Fernández Á.L., Rebollo-Roldán J., Jiménez-Rejano J.J., Güeita-Rodríguez J. (2015). Strength-Duration Curves of the Common Fibular Nerve Show Hypoexcitability in People With Functional Ankle Instability. PM&R.

[71] Zagatto A.M., Dutra Y.M., Claus G., de Sousa Malta E., de Poli R.A.B., Brisola G.M.P., Boullosa D. (2022). Drop jumps improve repeated sprint ability performances in professional basketball players. Biol. Sport.

[72] Baudry S., Duchateau J. (2007). Postactivation potentiation in a human muscle: Effect on the rate of torque development of tetanic and voluntary isometric contractions. J. Appl. Physiol..

[73] Bigland-Ritchie B., Jones D.A., Hosking G.P., Edwards R.H.T. (1978). Central and Peripheral Fatigue in Sustained Maximum Voluntary Contractions of Human Quadriceps Muscle. Clin. Sci..

[74] Rodriguez-Falces J., Place N. (2018). Sarcolemmal membrane excitability during repeated intermittent maximal voluntary contractions. Exp. Physiol..

[75] Renaud J.-M., Ørtenblad N., McKenna M.J., Overgaard K. (2023). Exercise and fatigue: Integrating the role of K^+^, Na^+^ and Cl^−^ in the regulation of sarcolemmal excitability of skeletal muscle. Eur. J. Appl. Physiol..

[76] Juel C. (1988). Muscle action potential propagation velocity changes during activity. Muscle Nerve.

[77] Cairns S.P., Flatman J.A., Clausen T. (1995). Relation between extracellular [K^+^], membrane potential and contraction in rat soleus muscle: Modulation by the Na^+^-K^+^ pump. Pflügers Arch..

[78] Cairns S.P., Leader J.P., Higgins A., Renaud J.-M. (2022). The peak force-resting membrane potential relationships of mouse fast- and slow-twitch muscle. Am. J. Physiol. Physiol..

[79] Rocchi L., Brown K., Di Santo A., Smith H., Peterchev A.V., Rothwell J.C., Hannah R. (2025). Distinct impacts of sodium channel blockers on the strength–duration properties of human motor cortex neurons. Epilepsia.

[80] Hodgkin A.L., Huxley A.F. (1952). A quantitative description of membrane current and its application to conduction and excitation in nerve. J. Physiol..

[81] Catterall W.A. (2012). Voltage-gated sodium channels at 60: Structure, function and pathophysiology. J. Physiol..

[82] Xie Y.-F., Yang J., Ratté S., Prescott S.A. (2024). Similar excitability through different sodium channels and implications for the analgesic efficacy of selective drugs. eLife.

[83] Goldfarb M. (2011). Voltage-gated sodium channel-associated proteins and alternative mechanisms of inactivation and block. Cell. Mol. Life Sci..

[84] Vandael D.H.F., Ottaviani M.M., Legros C., Lefort C., Guérineau N.C., Allio A., Carabelli V., Carbone E. (2015). Reduced availability of voltage-gated sodium channels by depolarization or blockade by tetrodotoxin boosts burst firing and catecholamine release in mouse chromaffin cells. J. Physiol..

[85] Kang Y.-J., Clement E.M., Sumsky S.L., Xiang Y., Park I.-H., Santaniello S., Greenfield L.J., Garcia-Rill E., Smith B.N., Lee S.-H. (2020). The critical role of persistent sodium current in hippocampal gamma oscillations. Neuropharmacology.

[86] Hennings K., Arendt-Nielsen L., Andersen O.K. (2005). Breakdown of accommodation in nerve: A possible role for persistent sodium current. Theor. Biol. Med. Model..

[87] Mogyoros I., Lin C.S., Kuwabara S., Cappelen-Smith C., Burke D. (2000). Strength-duration properties and their voltage dependence as measures of a threshold conductance at the node of Ranvier of single motor axons. Muscle Nerve.

[88] Stern S., Agudelo-Toro A., Rotem A., Moses E., Neef A. (2015). Chronaxie Measurements in Patterned Neuronal Cultures from Rat Hippocampus. PLoS ONE.

[89] Rattay F., Paredes L., Leao R.N. (2012). Strength–duration relationship for intra- versus extracellular stimulation with microelectrodes. Neuroscience.

[90] Kullo I.J., Hensrud D.D., Allison T.G. (2002). Comparison of numbers of circulating blood monocytes in men grouped by body mass index (<25, 25 to <30, ≥30). Am. J. Cardiol..

[91] Romero-Corral A., Somers V.K., Sierra-Johnson J. (2008). Accuracy of body mass index in diagnosing obesity in the adult general population. Int. J. Obes..

[92] Mazic S., Djelic M., Suzic J. (2009). Overweight in trained subjects—Are we looking at wrong numbers? (Body mass index compared with body fat percentage in estimating overweight in athletes.). Gen. Physiol. Biophys..

[93] Santos D.A., Silva A.M., Matias C.N. (2015). Utility of novel body indices in predicting fat mass in elite athletes. Nutrition.

[94] Comfort P., Bullock N., Pearson S.J. (2012). A Comparison of Maximal Squat Strength and 5-, 10-, and 20-Meter Sprint Times, in Athletes and Recreationally Trained Men. J. Strength Cond. Res..

[95] Terzis G., Kyriazis T., Karampatsos G. (2012). Muscle Strength, Body Composition, and Performance of an Elite Shot-Putter. Int. J. Sports Physiol. Perform..

[96] Case M.J., Knudson D.V., Downey D.L. (2020). Barbell Squat Relative Strength as an Identifier for Lower Extremity Injury in Collegiate Athletes. J. Strength Cond. Res..

[97] Pürzel A., Kaufmann P., Koller W. (2025). Biomechanical analysis of hip, knee, and ankle joint contact forces during squats in elite powerlifters. PLoS ONE.

[98] De Oliveira B.L., Pfeiffer E.R., Sundnes J., Wall S.T., McCulloch A.D. (2015). Increased Cell Membrane Capacitance is the Dominant Mechanism of Stretch-Dependent Conduction Slowing in the Rabbit Heart: A Computational Study. Cell. Mol. Bioeng..

[99] Dougherty P.M., Chen J. (2016). Relationship of membrane properties, spike burst responses, laminar location, and functional class of dorsal horn neurons recorded in vitro. J. Neurophysiol..

[100] Boinagrov D., Loudin J., Palanker D. (2010). Strength–Duration Relationship for Extracellular Neural Stimulation: Numerical and Analytical Models. J. Neurophysiol..

[101] Rozand V., Grosprêtre S., Stapley P.J., Lepers R. (2015). Assessment of Neuromuscular Function Using Percutaneous Electrical Nerve Stimulation. J. Vis. Exp..

[102] Brümmer V., Schneider S., Strüder H., Askew C. (2011). Primary motor cortex activity is elevated with incremental exercise intensity. Neuroscience.

[103] Collins B.W., Gale L.H., Buckle N.C.M., Button D.C. (2017). Corticospinal excitability to the biceps brachii and its relationship to postactivation potentiation of the elbow flexors. Physiol. Rep..

